# On Field Infrared Thermography Sensing for PV System Efficiency Assessment: Results and Comparison with Electrical Models

**DOI:** 10.3390/s20041055

**Published:** 2020-02-15

**Authors:** Mirco Muttillo, Iole Nardi, Vincenzo Stornelli, Tullio de Rubeis, Giovanni Pasqualoni, Dario Ambrosini

**Affiliations:** 1 Department of Industrial and Information Engineering and Economics (DIIIE), University of L’Aquila, Piazzale Pontieri 1, Monteluco di Roio, I 67100, 67100 L’Aquila, Italy; mirco.muttillo@graduate.univaq.it (M.M.); tullio.derubeis@univaq.it (T.d.R.); giovanni.pasqualoni@univaq.it (G.P.); dario.ambrosini@univaq.it (D.A.); 2 ENEA-Italian National Agency for New Technologies, Energy and Sustainable Economic Development, 00123 Rome, Italy; iole.nardi@enea.it

**Keywords:** PV system, infrared thermography, electronic systems, electric efficiency, faults diagnostic

## Abstract

The evaluation of photovoltaic (PV) system’s efficiency loss, due to the onset of faults that reduce the output power, is crucial. The challenge is to speed up the evaluation of electric efficiency by coupling the electric characterization of panels with information gathered from module diagnosis, amongst which the most commonly employed technique is thermographic inspection. The aim of this work is to correlate panels’ thermal images with their efficiency: a “thermal signature” of panels can be of help in identifying the fault typology and, moreover, for assessing efficiency loss. This allows to identify electrical power output losses without interrupting the PV system operation thanks to an advanced PV thermography characterization. In this paper, 12 faulted working panels were investigated. Their electrical models were implemented in MATLAB environment and developed to retrieve the ideal I-V characteristic (from ratings), the actual (operative) I-V characteristics and electric efficiency. Given the curves shape and relative difference, based on three reference points (namely, open circuit, short circuit, and maximum power points), faults’ typology has been evidenced. Information gathered from infrared thermography imaging, simultaneously carried out on panels during operation, were matched with those from electrical characterization. Panels’ “thermal signature” has been coupled with the “electrical signature”, to obtain an overall depiction of panels’ health status.

## 1. Introduction

The need for increasing the share of electric energy produced by renewable resources has pushed, over the last years, the installation of photovoltaic (PV) plants. According to statistics [1] less than 0.3% of the power and heat in the world is supplied by the solar PV. This share is low if compared to other renewables, therefore, a large amount of investment and support measures have been financed all over the world to incentivize this kind of source [1,2]. Some encouraging results are given by the installed power, which increased over the last decades, especially in some regions like Europe, to gain energy independency from unstable countries that might have unpredictable energy markets [3].

By now, given the age of installed systems, a new concern is becoming relevant: the evaluation of PV panels’ efficiency, that might decrease for the onset of faults (like delamination, defective bypass diode, cell breakage), and for materials aging [4,5].

The possibility of closely modelling the PV panels’ behavior is, in this perspective, crucial. This led to the development and proposal of several models and development and analysis, using electrical equivalent circuits that, according to studies [6], involve linear and non-linear components. These models allow to compare the standard I-V characteristics with the operative one. Despite the ease of approach, such comparison is quite complex: PV modelling requires the knowledge of specific circuit variables and environmental parameters normally supplied by acquisition systems [7,8,9,10,11,12,13]. Therefore, attention must be paid to the estimation of the elements involved in the model itself, in terms of components and variables, usually referred to as “model parameters”, like photocurrent, saturation current, diode ideality factor, and shunt and series resistances. 

This modelling and comparison, which is widely discussed in literature as a recent review shows [6], can be sided by on field diagnosis and evaluation of the PV plant. The challenge for industry insiders is to speed up the evaluation of electric efficiency of plants that might account for hundreds of modules, for instance, recurring to statistics [14], or to artificial neural networks [15]. 

Another way is coupling the electric characterization of panels with information gathered from diagnosis techniques, amongst which the most commonly employed is thermographic inspection [16,17,18].

In fact, through the thermal image, it is possible to identify local thermal anomalies and to hypothesize possible kind of fault, whilst, through the I-V characteristics, it is possible to infer the kind of fault (according to I-V curve) and to determine the electric losses.

Therefore, modelling and sensing imaging provide complementary information on the PV system. Many research efforts [19,20,21] have been devoted to link temperature patterns to fault typology and photogrammetric studies performed with aerial thermographic inspection [22,23] have become more popular.

However, at the best of authors’ knowledge, a paper that simultaneously considers panel ideal characteristics, the operative I-V curve, and the correlation between outdoor thermal images of panels and their efficiency, is still missing. A preliminary approach, considering single point measurements has been described by the same authors in a national conference [24]. The goal of this work is to have a “thermal signature” of panels, through which identify faults typology and, simultaneously, to assess the value or a range of values of efficiency loss. Also, this work wants to prove that IR sensory thermography application can be of help for the retrieval of technical information regarding PV panels during their operation. 

Sometimes some parameters are not supplied by the manufacturer especially on old panels. The coefficients that take into account the variation of the voltage and current based on the temperature variation are omitted. Indeed, the integration of thermography allows to derive these dated implant parameters that are important for simulation of the behavior in different conditions. Through the thermography it is possible to identify the panels that do not present thermal defects and take them as samples to obtain the coefficients. If the coefficients are supplied, the thermography also allows the evaluation of the degradation of the panels over the years, which is not the same for all panels.

The aforementioned aspects are needed to obtain a panel simulation that is as close as possible to the real behavior and then to understand any defects by comparing simulated and measured results. This approach allows one to define the thresholds for which the panel is declared defective to be replaced or still good even in the presence of defects.

**Figure 1:**
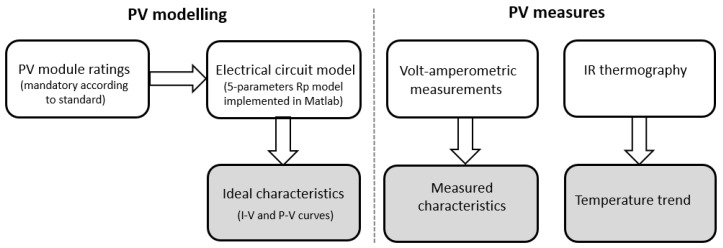
Employed methodology.

To carry out this study, 12 aged working panels have been investigated, following this procedure, also shown in Figure 1: (i) panels’ electrical model, implemented in the MATLAB(© 1994-2020 The MathWorks, Inc., Natick, MA, USA) environment, has been developed to retrieve the ideal I-V characteristic starting from ratings available from marks on panels; (ii) the actual I-V characteristics were retrieved after measuring electric current and voltage in a working day having environmental condition very close to that of STC (standard test conditions); (iii) ideal characteristics were compared to the measured ones to assess the differences induced by faults and aging. Given the curves shape and relative difference, based on three reference points (namely, open circuit, short circuit, and maximum power points), faults’ typology has been evidenced. Then, (iv) starting from the operative I-V characteristics, panels’ electric efficiency has been evaluated, and (v) compared to the expected one (obtained from ratings) and to the modeled one. Finally, (vi) information from thermal inspection were then matched with those from electrical characterization, for the retrieval of the “thermal signature”. 

For this reason, the paper is structured as follows: Section 2 describes the basics of PV fault diagnostics, both from the thermal and from the electric point of view, as a background for PV systems evaluation; Section 3 describes the experimental setup; and Section 4 and Section 5 show the results and their analysis, with final conclusions.

## 2. Basics on PV Faults Diagnostics

### 2.1. Thermal Approach

The need for PV plant diagnosis has led to the adoption of techniques and methods for a reliable inspection. Given the fact that several types of faults and damages to PV systems give rise to thermal effects on modules’ surfaces, the technique that employs the thermal detection is the most suitable. Indeed, the heat load is uniform for “healthy” cells, in which there is near-uniform current density. On the contrary, with panel defects the heat load is uneven, and it might accelerate the degradation processes of the modules. Such uneven heat load gives rise to uneven temperature distribution, which can be sketched with a device like an infrared camera.

Therefore, the infrared thermography (IRT) has been successfully applied for PV system diagnosis, since it entails some advantages, like contactless and nondestructive detection, fast inspection on large areas, and the continuity of operation of the PV system during the diagnosis phase [25,26]. 

Research efforts over the last decades have been devoted to the demonstration of the applicability of the technique in outdoor conditions [27], and to the correlation between thermal images pattern and fault typology [28].

In 2012, a review on thermography applied to photovoltaic systems [29] was published, claiming that its employment began more than a decade before. This work outlines the factors that, according to previous literature, degrade PV modules (i.e., degradation of packing materials; loss of adhesion of encapsulant; degradation of cell/module interconnection or of semiconductor device; moisture intrusion). Then, a focus is dedicated to those faults, which are generally classified as “hot spots”, since they are revealed, in the thermal image, as points having higher temperature. The work proposes the description of such defects, together with a comparison of visible-thermal images of such defects.

The most comprehensive work in this field is probably [16], where the employment of IRT for fault detection is widely described and discussed, starting from a classification of fault types. Moreover, in [16], a match between fault type, thermal pattern, I-V curve shape, and possible electrical degradation is clearly summarized. This fault classification can be of help for the fast identification of fault typologies. 

### 2.2. Electrical Approach

For the analysis and modelling of photovoltaic panels it is necessary to recreate the I-V curve starting from the manufacturer’s rating plate parameters. These data are mandatory stamped on panels due to the standard EN 50380 [30], that requires the declaration of *V_oc_*, *V_MPP_*, *I_MPP_*, *P_MPP_,* and *I_sc_* under standard test conditions (STC), which correspond to a solar irradiance of 1000 W/m^2^ with 1.5 air mass spectrum at temperature 25 °C and after stabilization. 

Thanks to the equivalent circuit models of the PV cells, it is possible to simulate their behavior using standard software, such as MATLAB or PSpice. Indeed, with these models, it is possible to simulate the single cell, the complete panel, or the whole plant. 

In the literature, according to a recent review [6], four main equivalent models are most commonly employed: (i) the ideal single diode model; (ii) the single diode Rs-model; (iii) the single diode Rp-model; and (iv) the two diodes model. These models apply differently according to the required I-V characteristic: for instance, the ideal single diode model (that has three parameters, namely *I_PV_*, *I_0_*, and *a*) does not take into account p-n junction losses, whilst the single diode Rs- model does.

The Rp-model (whose parameters are *I_PV_*, *I_0_, a*, *R_s_*, and *R_p_*) takes also into account the temperature dependence of the shunt resistance. Finally, the two diodes model increases the number of parameters (from 5 to 7), but reduces the simulation time and has better accuracy in the case of low solar irradiance.

In this work, the Rp-model has been employed, since it combines the ease of modelling with precision [31,32], and, moreover, is available in the MATLAB environment [33]. 

**Figure 2:**
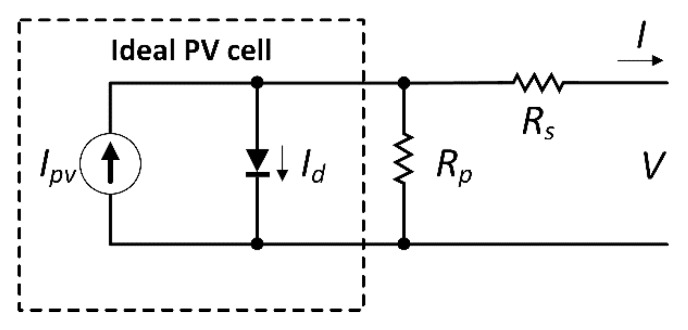
Equivalent circuit of the Rp-model.

The equivalent circuit of Rp-model is shown in Figure 2.

The current generated by the photovoltaic cell, in the presence of irradiance, is represented by a current source *I_pv_*. The flux of incident irradiance modifies the *I_pv_* magnitude, which also depends on the semiconductor material absorption capability. Furthermore, the circuit has a diode and a parallel resistor *R_p_* (shunt resistor), that respectively mimic the electron-hole recombination and the leakage current. The output current (I) can be expressed as:

(1) $${I\;=\;I}_{pv}-I_{0}\left[e^{\left(\frac{q}{\mathrm{akT}}\right)\left({V\;+\;R}_{s}I\right)}\;-\;1\right]-\frac{{V\;+\;R}_{s}I}{R_{p}}$$

In Equation (1), *a* is the ideality factor of the diode and the *I_0_* represents his saturation current. The electron’s charge (−1.60217646 × 10^−19^ C) is represented with constant *q*, *k* is the Boltzmann’s constant (−1.380653 × 10^−23^ J/K) and *T* is the junction’s temperature (expressed in K). Equation (1), which describes a single cell, can be generalized for *N* cells in series as follows:

(2) $${I\;=\;I}_{pv}-I_{0}\left[e^{\left(\frac{q}{\mathrm{akTN}}\right){(V\;+\;R}_{s}I)}-1\right]-\frac{{V\;+\;R}_{s}I}{R_{p}}$$

**Figure 3:**
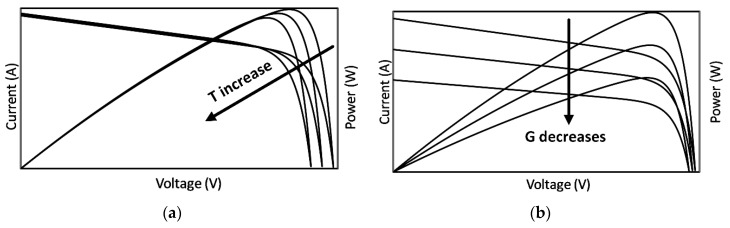
I-V and P-V curve. (**a**) at 1000 W/m^2^ and varying the panel’s temperature (**b**) at 25 °C and varying solar irradiance.

Typical I-V and P-V curves of Rp- model in various operative conditions (temperature and solar irradiance) are shown in Figure 3. In detail, Figure 3a shows the curves shift when temperature increases at a given solar irradiance, and Figure 3b shows the curve shift when solar irradiance decreases at a given air temperature.

Add to this that PV panels also exhibit sensitivity to temperature increases [34].

Particularly, the dependence of the open circuit voltage *V_oc_* from the temperature is given by a coefficient provided by the manufacturer [35]. This coefficient *K_v_* (in %/°C) is used for calculation of the *V_oc_* by the following equation:

(3) $$V_{ocT}{=V}_{oc}{[1-K}_{v}{(T}_{panel}-25)]$$

where *T_panel_* is the panel’s temperature.

The aging-related degradation of panel leads to the decline in ‘optical’ performance and to a reduction in the estimated power, which can vary from panel to panel depending on the type and year of production. Sometimes this value is provided by the manufacturer, and it can be used to evaluate the natural decrease in the performance of that specific panel; in the absence of specific information, the average value of 0.8% [36,37] can be used (as has been done in [16]).

This degradation mainly impacts on the shot and maximum current, but negligibly affects the output voltage [16]. Therefore, the characteristic curve can be corrected by appropriately reducing only the current values and keeping the voltage range unchanged.

**Figure 4:**
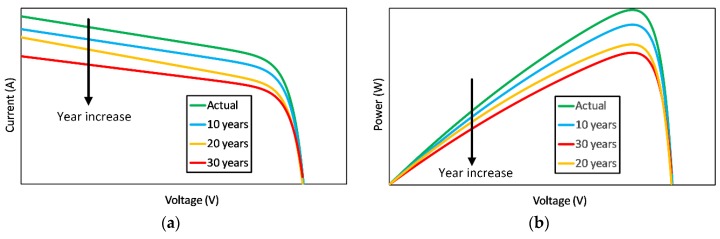
Correction of the characteristic curve based on aging (10, 20, and 30 years). (**a**) I-V characteristic; (**b**) P-V characteristic.

The modified value of short-circuit and the maximum power current to generate the aged I-V characteristic are calculated through Equations (4) and (5), respectively:

(4) $$I_{SCa}{=I}_{sc}\{1-[r\left(AY-IY\right)]\}$$

(5) $$I_{MPPa}{=I}_{MPP}\{1-[r\left(AY-IY\right)]\}$$

where *AY* is the actual year and *IY* is the installation year. Typical trends of I-V and P-V curves corrected for aging (after 10, 20, and 30 years from the installation date) considering a power reduction rate (*r*) of 0.8% per year are shown in Figure 4a,b.

Finally, it is necessary to model the panels’ electrical efficiency, which can be expressed by Equation (6), proposed in [38]:

(6) $$\eta \;=\;\frac{V_{OC}I_{SC}FF}{GA}100$$

where *FF* is the fill factor, *G* is the solar irradiance (W/m^2^), and *A* is the total area of PV panel (m^2^).

The reduction in the power output of a PV plant, caused by the factors that influence their performance, can have severe economic consequences [39].

For this reason, the knowledge of recurring degradation causes and their effects is crucial, since it can help to reduce the extraordinary maintenance cost, due for instance to the substitution of broken/nonworking panels. To partially solve the problem, several monitoring systems have been conceived [40,41]. Such systems allow to intervene before breakage and damages, but entail maintenance costs.

The literature of the last decade has evidenced the most recurring types of fault that might affect the PV modules functionality [42,43,44,45]. It is worth noting that fault refers to a specific condition that can alter the work of a panel, thus reducing the output power, and can induce consequences with respect to safety risks.

For this reason, fouling, dust, leaves, and bird droppings are not considered as faults, although they shadow the panels, thus reducing their output [46].

**Figure 5:**
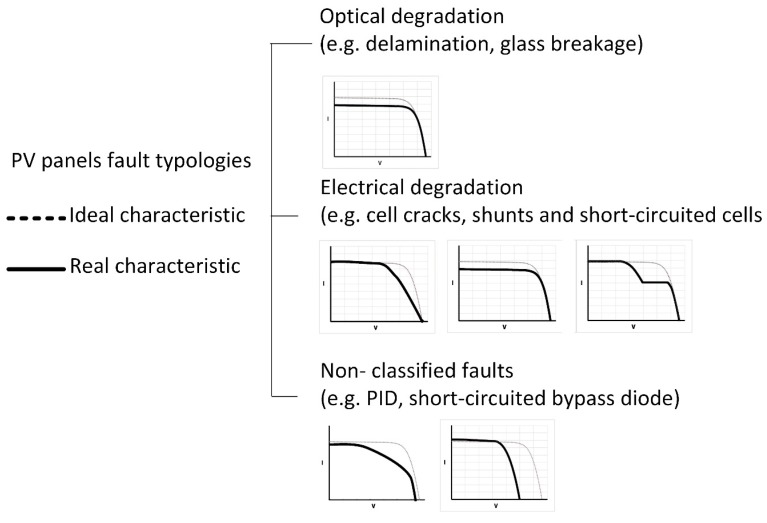
Fault typologies and the real I-V characteristic (adapted from [16]).

Faults can be divided into three main categories, according to the classification proposed in [16] and summarized in Figure 5. Such faults differently alter the ideal I-V characteristic. Therefore, by retrieving the real I-V curve, and by comparing it with the ideal one, it is possible to infer possible fault typologies.

This, of course, is possible in the case of on-site monitoring.

However, the knowledge of other kinds of degradation factors that might have altered the panels behavior are also necessary, above all the aging of the panels.

To take into account the time passed from the installation to the actual date, some aging factors can be considered, as proposed in [16,40], and following simple correlations like those of Equations (3) and (4).

## 3. Experimental Setup

For the purpose of this work, 12 panels were used. Each panel has two series of 18 cells each, giving an overall number of cells equal to 36. Such modules, installed on a metal frame structure on a flat roof of the University of L’Aquila (L’Aquila, Italy; 42° 20’ 15.896" N 13° 22’ 37.240" E) in 1990, have been scarcely used unless for research purposes, but they have been exposed to natural weathering during their lifetime. 

Electrical and thermal measurements were made in two days: the first day was August 28th and the second August 29th. The first electrical and thermal measurements were taken on August 28th on all panels. During the second day of measurements, five panels called as P5, P6, P10, P11, and P12 have been modified. In the panels P5, P6, P11, and P12 the bypass diodes have been replaced and in panel P10 a diode break has been simulated by disconnecting a string. For this reason, thermal measurements were taken on all the panels and electrical measurements were taken on only 6 panels which are P4, P5, P6, P10, P11, and P12. P4 was not modified but electrical measurements were made over the two days.

For the thermal measurements all the panels have been connected in short circuit. The I-V characteristics of the panels were measured immediately after the end of the thermal measurement campaigns. Therefore, the temperatures used for the simulations are those of the last frame of the thermal measurements, assuming that they do not vary so much.

Furthermore, the first test is used to derive the *K_v_* coefficient, not provided by the manufacturer, through Equation (3).

The panels’ datasheets were no longer available, but ratings marked on their back were sufficient for the modelling. 

### 3.1. Thermographic Setup

To retrieve the panels’ “thermal signature”, an IR camera (type FLIR S65H, FPA uncooled microbolometric sensor, 320 × 240 pixels, FOV 24° × 18°, working in the spectral range 7.5–13 μm, measuring range −40–1500 °C, resolution 0.05 °C, IFOV equal to 1.1 mrad, and error at 25 °C equal to ±2 °C or ±2% of the range) was employed. This equipment operates in the infrared atmospheric window where the air transmittance is higher.

Thermal images were acquired with a rate of 10 seconds during the panels’ heating phase, which was due to the solar exposure and electric load application. A tripod, placed 7 m from the panels, allowed continuous acquisition. The distance was chosen in order to:

- view all the panels simultaneously; and
- avoid the possibility for the IR camera to influence the acquisition.

**Figure 6:**
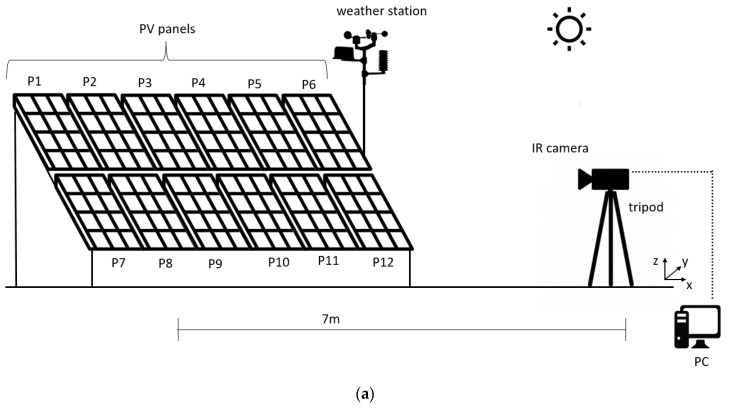

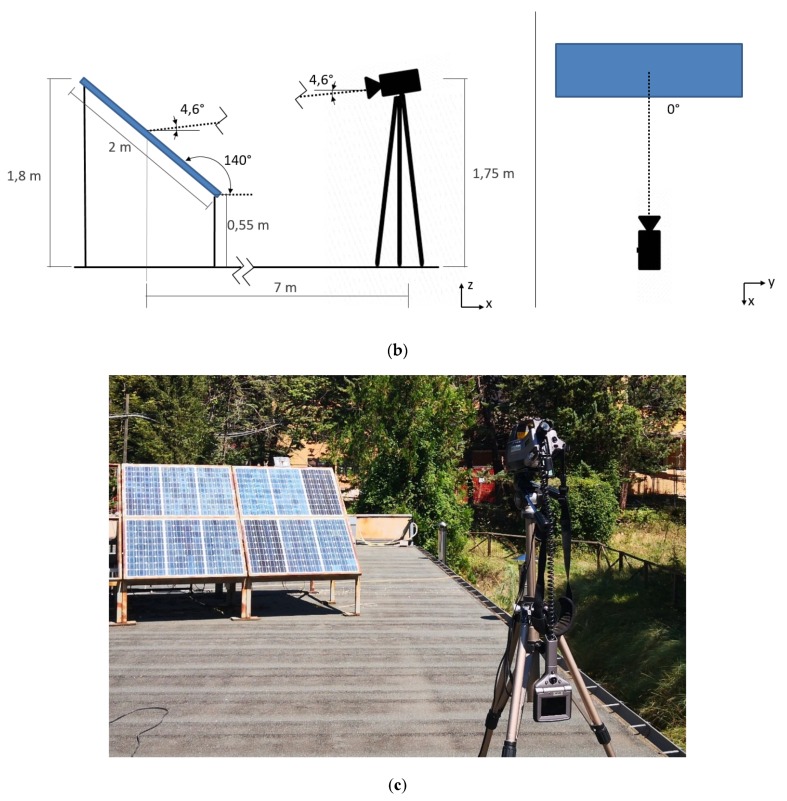
(**a**) Schematic of the experimental setup for thermal imaging; (**b**) thermal camera layout with respect to the panels (drawing is not to scale); (**c**) photograph of the setup.

For this reason, the viewing angle (the angle between IR camera lens and the perpendicular to the investigated object) was 0°. Therefore, the thermal camera was placed in front of the panels to perfectly acquire all of them in a symmetrical way (Figure 6b). The IR camera was slightly tilted (4.6°) to perfectly frame all the panels simultaneously. This is a bit in contrast with the common practice, where such viewing angle is discouraged due to the reflection of the camera itself. However, given the distance between the tripod and the PV panel, and their tilt angle with the ground, there was no possibility of self-reflection. This is also proved by the thermal image shown in Figure 8: if there had been a self-reflection, it would be seen in the thermal image.

A schematic of the experimental set up is drawn in Figure 6a,b and a photograph of the setup is shown in Figure 6c.

Thermal images, acquired from 12:00 a.m to 12:29 a.m on the first day, and from 12:30 a.m. to 1:10 p.m. the second day, were post-processed with specific software (ThermaCam Researcher). The choice of the start and end of the monitoring period was made according to the expected global solar radiation peak. For the August 28th, the peak was expected at 12:30, therefore, the acquisition was stopped just before this time. Unexpectedly, the solar radiation continued to increase for the following 30 minutes. However, this information has been available in the evening, when data were processed. Therefore, taking into account this detail, for the second day of acquisition (29th of August), the start was planned 30 minutes later. In this case, the peak was perfectly reached, and soon after the global solar radiation began to drift. In any case, the campaigns were carried out close or in correspondence to the maximum solar radiation, when the panels could have reached the highest temperatures. The measured panels’ thermal emissivity is 0.855. 

The panels mean temperature profile along time has been monitored during the two experimental campaigns: the results are shown in Section 4.

### 3.2. Electrical Setup

**Figure 7:**
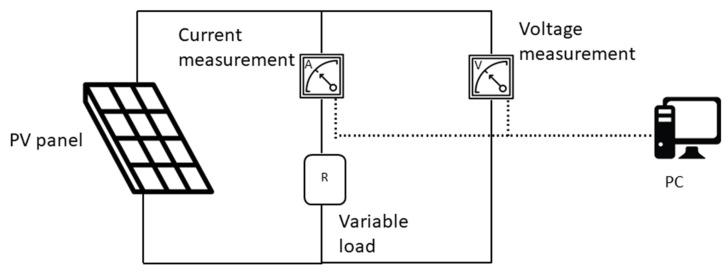
Experimental setup of electrical measurements.

Measurements were carried out during a sunny day, with an average irradiance of 1000 W/m^2^ measured by a pyranometer (DPA053 and M-Log Mini datalogger) placed nearby. To carry out volt-amperometric measurements, needed to reconstruct the characteristic curves of the panels, two multimeters (Agilent 34401A) connected to a PC were employed on a variable load, according to the setup shown in Figure 7.

Current and voltage were measured after modifying the resistance load, at fixed values. Data, stored in a PC, were then processed and compared with the simulation output.

Results are shown in Section 4.

## 4. Results

### 4.1. Thermograpfic Inspection Results

**Figure 8:**
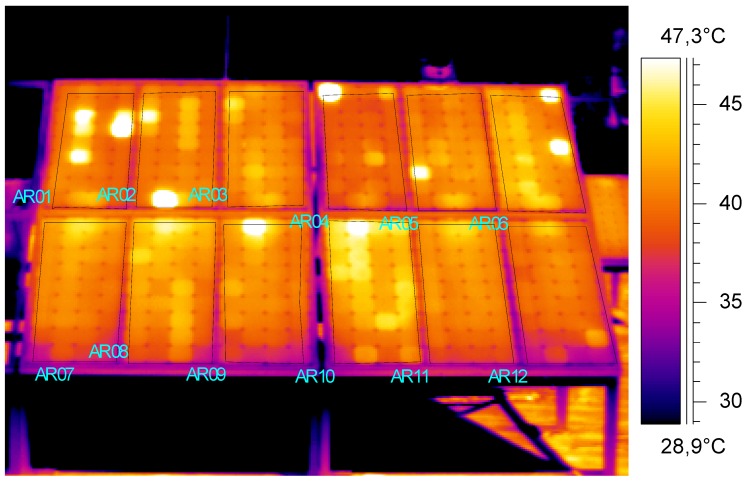
Example of an IR image of the investigated panels.

The setup employed for the thermographic inspection allowed the acquisition of two series of IR images (one for each campaign), like the one shown in Figure 8.

Panels’ temperature pattern variations with time due to the heating have been qualitatively analyzed. This entails the qualitative evaluation of local temperature anomalies or variations, in order to assess possible faults, given the fault classification table provided in [16].

Moreover, the panels’ mean temperature profile with time has been quantitatively analyzed; this step includes the plot, with time, of the average temperature of panels’ surfaces, thus excluding the bearing frames. For this purpose, 12 areas have been drawn into the interface of the software ThermaCAM Researcher, clearly visible in Figure 8. Such areas (namely, AR1, AR2, …, AR12) follow the panels’ numbering shown in Figure 6a.

From the temperature pattern analysis, it is possible to suppose that P1 suffers from the short-circuiting of bypass diodes, and panels P2, P4, and P9 might have optical degradation from broken cells, whilst P6 and P10 might have defective bypass diodes or internal short-circuits.

**Figure 9:**
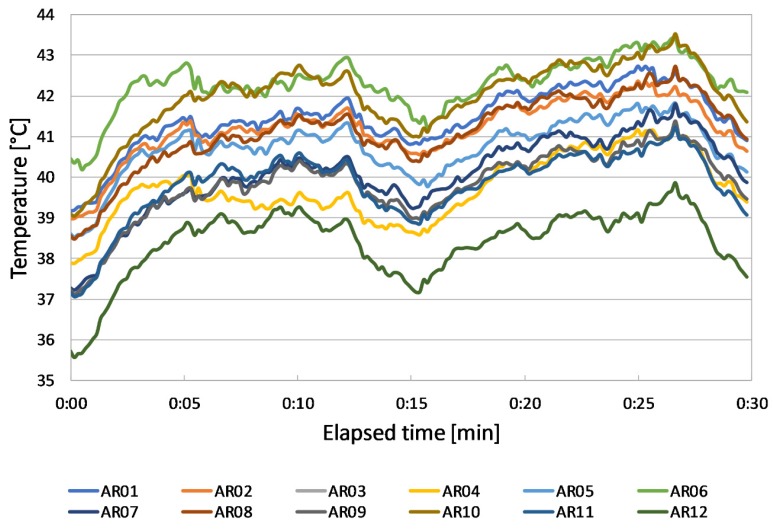
Panels’ temperature profiles during Test 1 (28th August).

Temperature profiles of Figure 9 are quite similar but shifted from each other. The lowest temperature values are reached by panel P12, which also shows the more even temperature pattern from the qualitative point of view.

The highest temperatures are alternatively reached by P6 and P10, followed by P1, P2, P8, P3, and P5.

Panels P7, P9, P4, and P11 show similar temperatures in the second half of the campaign, whilst they have oscillating trends in the first part.

**Figure 10:**
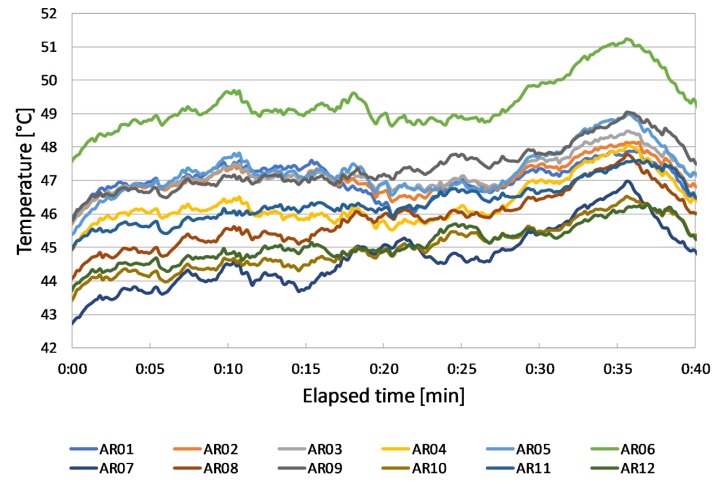
Panels’ temperature profiles during test 2 (29th August).

During test 2, temperature profiles shown in Figure 10 are recorded.

Once again, P6 has the highest temperatures, which differ by 2 °C on average from the temperature of P10.

**Figure 11:**
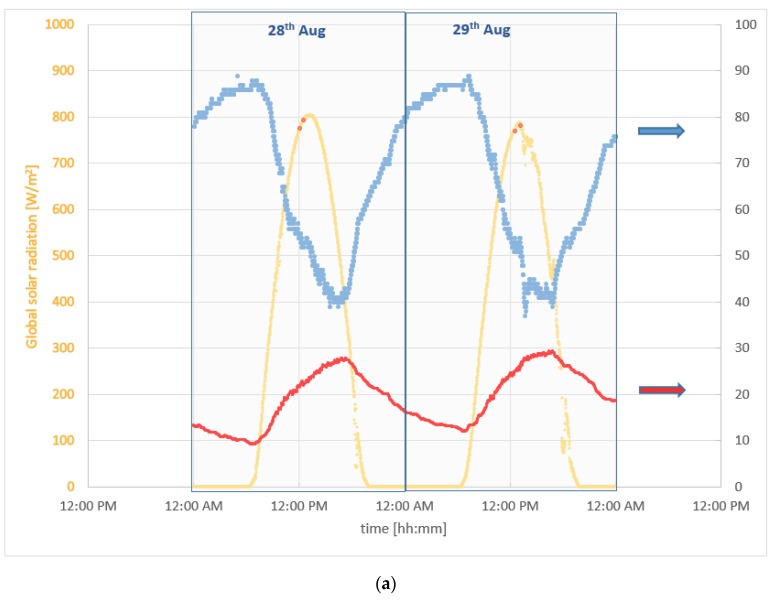

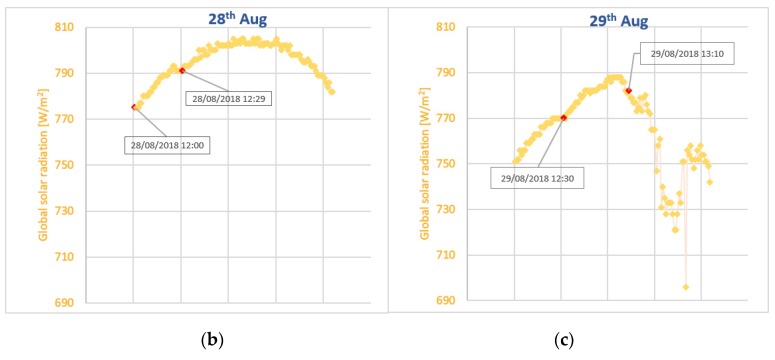
(**a**) Global solar radiation (left axis), outdoor temperature and relative humidity (right axis) recorded during the experimental campaigns. Red dots on the global solar radiation plot mark the start and end of the two thermographic campaigns; (**b**) global solar radiation between 12:00 a.m. and 2 p.m. of 28th August; (**c**) global solar radiation between 12:00 a.m. and 2 p.m. of 29th August.

The global solar radiation and the outdoor temperature have been recorded by a weather station placed in the city; the data are shown in Figure 11.

### 4.2. Model and Electrical Results

The I-V and P-V characteristics of the panels have been simulated and measured for defect characterization. Unfortunately, the data provided by the manufacturer for the panels are insufficient for simulation because there is no coefficient of reduction of the open-circuit voltage when the temperature varies. Thanks to thermography it is possible to derive this coefficient through Equation (3) on panel 7, which results from the thermal analysis without defects. In fact, by measuring the *V_oc_* and the temperature of the panel with IR camera, which is 18.85 V at 39.6 ° C, the coefficient is equal to 0.91%/°C.

Once the coefficient of the open-circuit voltage is determined based on the temperature, the short-circuit and maximum currents are calculated using Equations (4) and (5). As the panels have a life of 30 years the new current values are shown in Table 1.

When all the necessary values have been found, the IV characteristic of the panels is measured. The IV characteristic is measured for each panel and the solar irradiance is measured by means of a pyranometer with the same inclination and position of the group. The superficial temperatures are taken from the last frame of the measurements by the thermal camera.

With this data collected and the new calculated data, the data is passed to the simulation of the panels. In Table 2 the rating, simulation, and experimental results are shown. Panel simulations are performed using the solar irradiances and temperatures measured during the two tests. 

The difference between simulated and measured values allows one to determine a threshold on the measured values to detect the damage of the panels. The parameters to be examined are the short-circuit current, the open-circuit voltage, the maximum power point, and the efficiency. These differences between the simulated and measured values deriving from the first and second tests are shown in Table 3 and Table 4.

• Panel 1:

From the first test by the simulation and measurement results the short-circuit current decreases by 0.350 A with respect to the expected value. The short-circuit current is the only parameter that differs much from the expected value, therefore, from an electrical analysis, it can be stated that it most likely presents optical degradation or electrical degradation. 

• Panel 2:

In the first electrical test has only a deviation of 0.108 A from the simulated value, lower than the difference of the panel 1. Therefore, panel 2 may have defects of optical degradation or electrical degradation type. 

• Panel 3:

In the first electrical test has two parameters that move from the expected value, namely the *I_sc_* and the *P_max_*. Thus, they present defects of optical degradation or electrical degradation and the measured efficiency is not very far from that expected.

• Panel 4:

All tests carried out showed high values for all the parameters; in particular, the measured efficiency in the first test is 53.6% percent lower compared to the modeled one.

• Panel 5:

The panel 5 has the open circuit voltage much lower than expected, therefore there are presence of an oxidized connection. Both tests find this defect with old and new bypass diode.

• Panel 6:

In the first test, there is a noticeable difference in efficiency and open circuit voltage. In the second test, the bypass diodes were replaced, improving the short-circuit current but leaving the other parameters unchanged. It can be said that the defects are due to cells or optical imperfections.

• Panel 7:

It was used as a reference to calculate the open circuit voltage decrease coefficient with respect to temperature. Only the measurements in the first test were made on this panel. It has no apparent electrical defect.

• Panel 8:

It was measured only in the first test where there was a difference in the short-circuit current of 5.4% and an efficiency difference of 5.2%. Probably it presents not very relevant optical or electrical degradation.

• Panel 9:

In the first test a 7.3% reduction compared to the expected value of the efficiency has been found. The other values are excellent so there is electrical degradation.

• Panel 10:

In the first test there was a high difference in yield and short circuit current. In the second test the diode rupture was simulated omitting a panel string and the result of the measurements brought an open circuit voltage difference of 46.4% and a 50.3% efficiency decrease. However, the short-circuit current passes from 9.2% to −2%.

• Panel 11:

In the first test efficiency and short-circuit current of 6.8% and 6.4% are found. In the second test the bypass diodes were replaced bringing the efficiency to 1.1% and the current to 0.5%. Thus, having replaced the diodes leads to an increase in the efficiency of the panel.

• Panel 12:

The same as panel 11. In the first test the differences that are solved by replacing the bypass diodes.

**Figure 12:**
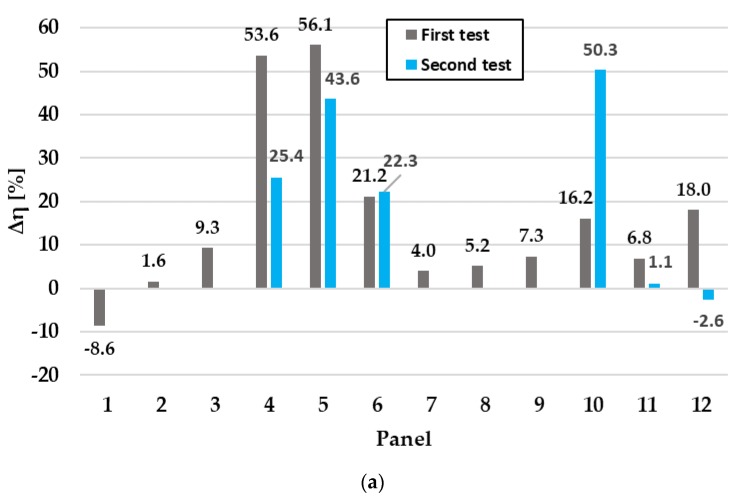

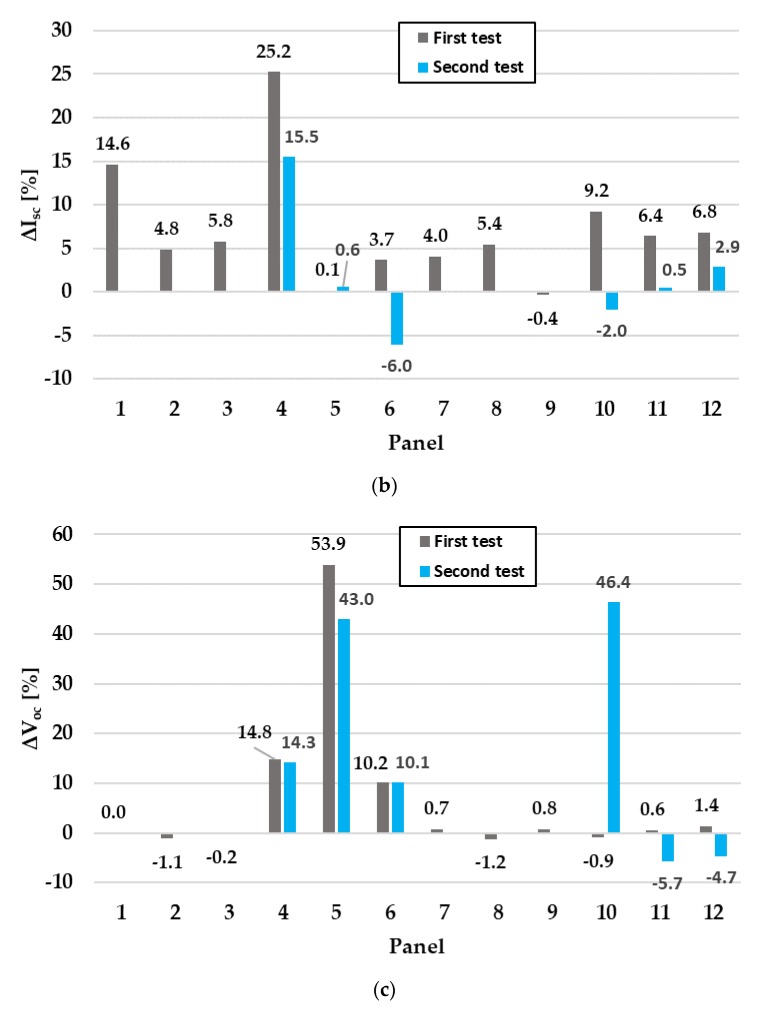
Percentage difference between expected simulated value and measured value for each panel: (**a**) efficiency; (**b**) short-circuit current; (**c**) open-circuit voltage.

The results with percentage variations of the investigated parameters are shown in Figure 12.

## 5. Conclusions

In this paper a methodology to analyze the behavior of photovoltaic panels by thermal and electrical analysis has been presented. To carry out this study, 12 aged working panels have been investigated. An electrical model has been implemented in MATLAB to retrieve the ideal I-V characteristic starting from ratings value provided by the manufacturer. The I-V simulated characteristic were compared with measured electrical current and voltage outdoors, with environmental condition very similar to that of STC. This comparison led to assess the differences induced by defects and aging. Given the curves shape and the relative difference, based on three reference points (namely, open circuit, short circuit and maximum power points), faults’ typologies have been evidenced. Then, panels’ electric efficiency has been evaluated, and compared to the expected one (obtained from ratings) and to the simulated one. Finally, the results from the thermal inspection were then combined with those of the electrical characterization and a defect analysis for each panel was described.

## Figures and Tables

**Table 1:** Values of short-circuit and maximum aging current calculated for each panel.

	Panel											
	1	2	3	4	5	6	7	8	9	10	11	12
*I_sc_* (A)	2.15	2.14	2.09	2.08	2.18	2.31	2.09	2.08	1.95	2.08	2.13	2.09
*I_max_* (A)	1.81	1.82	1.75	1.51	1.74	1.72	1.50	1.66	1.39	1.82	1.58	1.64

**Table 2:** Rating, simulation, and experimental results of each panel in the two tests.

Panel	Irradiance (W/m^2^)			Temperature (°C)			*V_max_* (V)			*I_max_* (A)			*P_max_* (W)			*I_sc_* (A)			*V_oc_* (V)			*FF* (-)			*η* (%)		
	S	M	R	S	M	R	S	M	R	S	M	R	S	M	R	S	M	R	S	M	R	S	M	R	S	M	R
1	1042	1042	1000	40.8	40.8	25.0	9.8	10.5	11.7	1.6	1.7	2.5	16.2	17.6	29.2	2.4	2.1	2.9	17.6	17.6	20.5	0.4	0.5	0.5	4.3	4.7	8.1
2	1032	1032	1000	40.6	40.6	25.0	12.1	12.3	14.8	1.8	1.8	2.5	22.3	21.9	36.9	2.2	2.1	2.9	18.0	18.2	21.0	0.6	0.6	0.6	6.0	5.9	10.3
3	1046	1046	1000	40.2	40.2	25.0	13.3	13.4	15.9	1.8	1.6	2.4	24.2	21.9	38.1	2.2	2.1	2.9	17.8	17.9	20.6	0.6	0.6	0.6	6.4	5.8	10.6
4.1	1048	1048	1000	39.3	39.3	25.0	17.9	16.6	19.1	1.8	1.0	2.1	32.3	16.6	39.5	2.2	1.6	2.9	22.1	18.8	21.7	0.7	0.5	0.7	9.5	4.4	12.4
4.2	949	949	1000	46.5	46.5	25.0	17.4	13.6	19.1	1.6	1.4	2.1	28.5	19.3	39.5	2.0	1.7	2.9	21.5	18.4	21.7	0.7	0.6	0.7	7.6	5.6	12.4
5.1	1061	1061	1000	40.0	40.0	25.0	14.4	6.4	17.1	1.8	1.8	2.3	25.9	11.4	39.1	2.2	2.2	2.9	18.5	8.6	21.4	0.6	0.6	0.6	6.8	3.0	10.9
5.2	955	955	1000	47.1	47.1	25.0	13.0	7.1	17.1	1.6	1.7	2.3	21.0	11.9	39.1	2.0	2.0	2.9	17.0	9.7	21.4	0.6	0.6	0.6	6.1	3.5	10.9
6.1	1057	1057	1000	41.7	41.7	25.0	13.0	14.4	17.3	2.2	1.6	2.3	28.4	22.4	39.1	2.5	2.4	3.0	20.3	18.2	21.0	0.6	0.5	0.6	7.5	5.9	10.3
6.2	970	970	1000	49.1	49.1	25.0	15.3	13.4	17.3	1.9	1.7	2.3	28.5	22.2	39.1	2.3	2.4	3.0	19.6	17.6	21.0	0.6	0.5	0.6	8.2	6.4	10.3
7	1036	1036	1000	39.4	39.4	25.0	14.4	14.4	17.0	1.6	1.5	2.1	23.1	22.2	35.1	2.2	2.1	2.9	19.0	18.8	21.7	0.6	0.6	0.5	6.2	6.0	9.7
8	1023	1023	1000	40.7	40.7	25.0	13.6	14.1	16.4	1.7	1.6	2.3	23.2	22.0	37.1	2.2	2.0	2.9	18.3	18.6	21.3	0.6	0.6	0.6	6.3	6.0	10.3
9	1036	1036	1000	39.4	39.4	25.0	14.0	13.4	16.3	1.5	1.4	1.9	20.8	19.2	31.0	2.1	2.1	2.7	18.8	18.6	21.3	0.5	0.5	0.5	5.6	5.2	8.6
10.1	1041	1041	1000	41.1	41.1	25.0	13.3	12.9	16.2	1.8	1.6	2.4	24.1	20.2	38.8	2.2	2.0	2.9	18.1	18.2	21.2	0.6	0.6	0.6	6.4	5.4	10.8
10.2	958	958	1000	47.4	47.4	25.0	12.2	6.5	16.2	1.7	1.5	2.4	20.2	10.0	38.8	2.0	2.1	2.9	16.8	9.0	21.2	0.6	0.5	0.6	5.8	2.9	10.8
11.1	1027	1027	1000	39.0	39.0	25.0	14.7	14.4	17.2	1.7	1.6	2.2	24.4	22.7	37.0	2.2	2.1	2.9	18.9	18.8	21.6	0.6	0.6	0.6	6.6	6.1	10.3
11.2	944	944	1000	45.9	45.9	25.0	13.3	13.5	17.2	1.5	1.5	2.2	20.5	20.3	37.0	2.0	2.0	2.9	17.4	18.4	21.6	0.6	0.5	0.6	6.0	6.0	10.3
12.1	1016	1016	1000	37.4	37.4	25.0	14.5	13.1	16.7	1.7	1.5	2.3	24.4	20.0	37.7	2.2	2.0	2.9	19.2	18.9	21.7	0.6	0.5	0.6	6.7	5.5	10.5
12.2	955	955	1000	44.7	44.7	25.0	13.1	13.9	16.7	1.6	1.5	2.3	20.8	21.4	37.7	2.0	2.0	2.9	17.7	18.5	21.7	0.6	0.6	0.6	6.1	6.2	10.5

Legend: (S) simulated; (M) measured; (R) rating; X.1: test 1 (28th August); X.2: test 2 (29th August)

**Table 3:** Differential values between simulation and measured results for the first test.

Panel	*ΔI_sc_* (A)	Δ*V_oc_* (V)	*ΔP_max_* (W)	*ΔFF*	*Δη* (%)
1	0.350	0.005	−1.397	−0.104	−0.369
2	0.108	−0.198	0.350	−0.013	0.098
3	0.128	−0.043	2.229	0.024	0.597
4	0.553	3.273	15.741	0.130	5.079
5	0.003	9.992	14.492	0.028	3.796
6	0.090	2.070	6.028	0.051	1.583
7	0.088	0.135	0.918	−0.004	0.246
8	0.118	−0.220	1.184	0.005	0.325
9	−0.008	0.151	1.511	0.037	0.406
10	0.202	−0.171	3.888	0.052	1.039
11	0.141	0.112	1.667	−0.001	0.448
12	0.147	0.261	4.410	0.064	1.204

**Table 4:** Differential values between simulation and measured results for the second test.

Panel	*ΔI_sc_* (A)	*ΔV_oc_* (V)	*ΔP_max_* (W)	*ΔFF*	*Δη* (%)
4	0.308	3.059	9.261	0.046	1.921
5	0.011	7.302	9.174	0.003	2.671
6	−0.137	1.985	6.352	0.119	1.821
10	−0.040	7.786	10.139	0.054	2.939
11	0.009	−0.999	0.228	0.035	0.065
12	0.059	−0.826	−0.538	−0.005	−0.159

## Abbreviations

**Nomenclature**	
PV	Photovoltaic
I	Current (A)
V	Voltage (V)
R	Resistance (Ω)
P	Power (W)
a	Diode Ideality factor (-)
STC	Standard test conditions
MPP	Maximum power point
k	Boltzmann’s constant (−1.380653 × 10–23 J/K)
T	Junction’s temperature (K)
N	Number of cell
q	Electron’s charge (−1.60217646 × 10–19 C)
FF	Fill factor (-)
PID	Potential induced degradation
G	Solar irradiance (W/m^2^)
AY	Actual year
IY	Installation year
A	Total area of PV panel (m^2^)
IR	Infrared
r	Power reduction rate
η	Panel efficiency
Δη	Panel efficiency drop
K	Temperature coefficient
**Subscripts**	
oc	Open circuit
sc	Short circuit
0	Diode saturation
p	Shunt
s	Series
d	Diode
v	Voltage
a	Aged
max	Maximum power point
